# Identification of *Streptococcus pneumoniae* *-*Specific Proteins by Surface-Shaving Proteomics

**DOI:** 10.1021/acs.jproteome.5c00716

**Published:** 2025-11-10

**Authors:** Leonarda Acha Alarcon, Guillem Seguí, Beatriz Piñeiro-Iglesias, Ema Svetlicic, Nahid Kondori, Margarita Gomila, Edward R. B. Moore, Roger Karlsson

**Affiliations:** † Department of Infectious Diseases, Institute of Biomedicine, Sahlgrenska Academy, University of Gothenburg, 40530 Gothenburg, Sweden; ‡ Centre for Antibiotic Resistance Research (CARe), 3570 University of Gothenburg, 40530 Gothenburg, Sweden; § Department of Clinical Microbiology, Sahlgrenska University Hospital, Region Västra Götaland, 41345 Gothenburg, Sweden; ∥ Culture Collection University of Gothenburg (CCUG), Sahlgrenska University Hospital and Sahlgrenska Academy, 41390 Gothenburg, Sweden; ⊥ Novo Nordisk Foundation Center for Biosustainability, Technical University of Denmark, 2800 Kongens Lyngby, Denmark; # Microbiology (Biology department), University of the Balearic Islands, Ctra. de Valldemossa km 7,5, 07122 Palma de Mallorca, Spain; ∇ Nanoxis Consulting AB, 40016 Gothenburg, Sweden

**Keywords:** *Streptococcus pneumoniae*, biomarkers, surface-shaving, proteomics, bottom-up mass spectrometry, LPI

## Abstract

*Streptococcus pneumoniae* (pneumococcus) is a prominent cause of bacterial pneumonia, meningitis, and septicemia, causing high morbidity and high mortality, particularly in children and the elderly. In this study, proteomics- and genomics-based approaches were used for the identification of pneumococcal protein and peptide biomarkers of *S*. *pneumoniae* for diagnostics and prospective targets for treatment. Through a pan-genome analysis, 11 *S*. *pneumoniae* strains, demonstrating genetic variation within the species, were selected for proteomic characterization. Mass spectrometry-based proteomics, in combination with bacterial surface*-*shaving, were used to study the cell-surface proteome of *S*. *pneumoniae*. The data obtained from three biological replicates per strain were analyzed to identify and rank the proteins and peptides according to their presence in the strains, as well as their presence in all available *S*. *pneumoniae* proteomes (8,892) archived in public databases. Several highly ranked proteins have been described as “species-specific” for *S*. *pneumoniae* and as surface-associated virulence factors or demonstrate highly antigenic properties. Proteins (34) previously not recognized as *S*. *pneumoniae*-specific were proposed to be novel biomarkers, demonstrating high degrees of prevalence in all analyzed proteomes, with little or no sequence similarities to closely related species but common among the genetically diverse strains included in this study.

## Graphical abstract

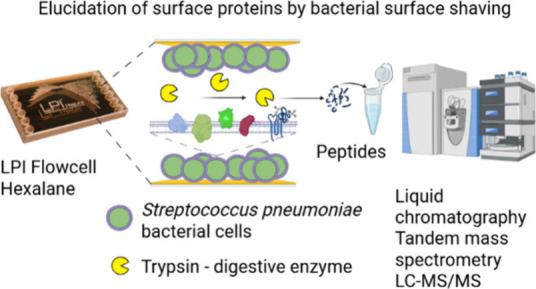

## Introduction

In 2019, infectious diseases caused approximately 13.7 million deaths;^1,2^ furthermore, infections caused by *Streptococcus pneumoniae* (pneumococcus) were ranked as the third highest cause of death,^1^ with high incidences of infections of the lower respiratory tract, blood, and meninges.^3–5^ More recently, in 2021, *S*. *pneumoniae* was described as the main bacterial cause of lower respiratory tract infections (97.9 million episodes) and related fatalities (505,000 deaths) around the world, a pattern that has persisted since 1990.^6^ Given its relevance, the World Health Organization (WHO) has included *S*. *pneumoniae* in the priority list of pathogens that have increased global health concerns.^3,7^

Traditional infectious disease diagnostics still rely on culture-based methods and isolation of pure colonies. These procedures are not only time-consuming but, in many cases, the isolation of viable bacteria or enough biomass for analyses are not possible,^8–11^ which may lead to low detection rates or, even, false negatives.^12^ For example, as many as 50% of *S*. *pneumoniae* sputum cultures have been observed to be reported as negative, even though it was shown that the patients had ongoing pneumococcal infections.^13^ Such observations highlight the importance of studying and understanding the molecular mechanisms behind *S*. *pneumoniae* infections,^3,14–17^ to improve the diagnostics and treatments of a pneumococcal infection.^5,18^

The increasingly lower cost and high throughput of Next-Generation Sequencing^3,10,19–21^ have enabled sequencing of thousands of *S*. *pneumoniae* strains,^22^ thus, facilitating biomarker discoveries by comparative genomics.^22,23^ For example, Kilian and Tettelin, 2019 described 224 genes specific for *S*. *pneumoniae* carbohydrate hydrolysis, capsule regulation, and proteins that help in immune evasion, which differentiate the species from closely related commensal bacteria, such as *Streptococcus pseudopneumoniae* and *Streptococcus mitis*.^24^

Even though genomic-based approaches have enabled comparative analyses between pathogenic and nonpathogenic strains; while also detecting traits of antibiotic resistance and virulence, genomics alone cannot validate the presence of putative biomarkers or strain-level traits. Recent developments in mass spectrometry-based proteomics have opened the capability of detecting the entire expressed proteome in a single analysis,^25,26^ and in combination with quantitative approaches, the relative abundance of interesting protein targets can be evaluated.^27,28^

The proteins at the surface of bacterial cells, or “surfaceome”, play key roles in host–pathogen interactions.^3,29–31^ Outer membrane proteins are surface-exposed and are crucial players in host–pathogen interactions, highly involved in virulence,^3,5,18,29–32^ enabling certain strains to become invasive.^14,33–35^ Surface proteins have been shown to be more variable in sequence, an important feature that enables the bacteria to evade the immune system or increase their virulence.^5,17,18,29–32,36,37^ Examples of such proteins are those related to metal binding or ion transporters like iron, zinc, and/or manganese metal ion transporters,^30,31,38^ which enables the bacteria to acquire necessary nutrition when infecting a host. In addition, proteins involved in cell adhesion, a key step in the infection process, have been shown to have changes in abundance when exposed to human cells.^33,39^ Furthermore, due to their surface exposure, the surface proteins are also interesting targets for antibody-based detection and development of rapid point-of-care diagnostics approaches.

Surface-shaving techniques, wherein proteins on the surface of intact cells are enzymatically digested into peptides, followed by tandem mass spectrometry analysis, enable the study of the surfaceome, including virulence factors, transporters, and immune evasion proteins exposed on the cell surface of bacteria.^33,39–43^ Lipid-based Protein Immobilization (LPI, Nanoxis Consulting AB, Gothenburg, Sweden) has been used to perform surface-shaving of intact bacteria of different species.^39,42,44–46^ The advantages of using the LPI methodology in a flow-cell format include the use of short and controlled enzyme digestion times, which enable the generation and identification of peptides from exposed proteins.

Here, intact bacteria from 11 clinical *S*. *pneumoniae* strains, exhibiting genetic variation within the species, were enzymatically shaved under controlled conditions to identify potential protein and peptide biomarkers that could be used for identification at the species level. Commonly detected species-unique peptide biomarkers, as well as species-unique proteins were identified and ranked according to their presence in the proteomes and genomes of the analyzed strains.

## Materials and Methods

### Comparative Genomic Analysis for Strain Selection

In November 2021, the genome sequences of *S*. *pneumoniae* strains available at the Culture Collection University of Gothenburg (CCUG, www.ccug.se) were retrieved from the National Center for Biotechnology Information (NCBI). Only genomes in the Genome Assembly database with a CCUG accession number were selected. As described by Gonzales-Siles et al., (2020)^46^ the taxonomic identity was verified, using Average Nucleotide Identity based on BLAST (ANIb) in JspeciesWS (https://jspecies.ribohost.com/jspeciesws), by comparing all genomes to each other, including the genome of the type strain *S*. *pneumoniae* ATCC 33400^T^ (CCUG 28588^T^), which was used as a reference. Additionally, Genome-to-Genome Distance Calculator (GGDC) was performed, using the GGDC Distance Calculator 3.0 (https://ggdc.dsmz.de/ggdc.php). The comparisons were calculated for 24 genomes. A total of 22 genomes are of *S*. *pneumoniae* strains and the genomes of the type strains of two closely related species, *S*. *pseudopneumoniae* (CCUG 49455^T^) and *S*. *mitis* (CCUG 31611^T^), were included in the analysis to confirm the strain taxonomy. The final data set consisted of unique pairwise values, obtained by averaging the values of the pairwise duplicates from each square matrix. Dendrograms were generated for each matrix to assess their phylogeny (data not shown). The *S*. *pneumoniae* strain characteristics, the strain accession numbers, the respective genome sequence accession numbers and their ANIb and GGDC scores against the *S*. *pneumoniae* type strain are listed in Supporting Information 1. The genomes were annotated, using Prokka v1.14.6,^47^ and a pangenome analysis was performed with Get_Homologues,^48^ as indicated in Gonzales-Siles et al., 2020.^46^ Based on the pangenome analysis, a phylogenetic tree was generated to observe the relationship among the strains. The strains, representing genomic variation (11) within the analyzed genomes, were selected for surface-shaving (Supporting Information 2).

### Strain Characteristics, Culture Conditions, and Optimization of Sample Preparation

The *S*. *pneumoniae* strains, CCUG 28588^T^, CCUG 1350, CCUG 6798, CCUG 17113, CCUG 32672, CCUG 33774, CCUG 35180, CCUG 35229, CCUG 63093, CCUG 69380, and CCUG 69382, all isolated from clinical samples, were obtained from the CCUG. The strains were cultivated on Columbia Agar Base with 5% (v/v) defibrinated horse blood (Substrate Department, Sahlgrenska University Hospital, Gothenburg, Sweden) at 36 °C in the presence of 5% CO_2_ for 18 h (stationary phase). Sample preparation conditions were optimized with *S*. *pneumoniae* strain CCUG 28588^T^. After incubation, bacterial biomass was resuspended in 1.0 mL of Phosphate Buffered Saline 0.01 M (PBS; 806552–1l, Sigma-Aldrich, Saint Louis, MO, United States) and centrifuged at 4,000*g* for eight min at 4 °C, repeating the washes with PBS three times. Optical Densities (OD) were measured at a wavelength of 600 nm and adjusted to OD 1.0, using a CO8000 Cell Density Meter (490005–906, WPA Biowave, Cambridge, United Kingdom). All strains were analyzed in three biological replicates.

### Surface Shaving and Generation of Peptides for LC-MS/MS Analysis

Surface shaving was used to generate peptides, following a variation of the method described by Wolden et al., 2020.^39^ Briefly, 50 μL of the bacterial suspensions were injected into the LPI Hexalane flow-cell channels (Nanoxis Consulting AB, Gothenburg, Sweden). The bacterial suspension was incubated in the flow-cell channels for 30 min at room temperature to promote immobilization of the intact cells onto the membranophilic surface.^42^ The excess, nonbound bacteria, were rinsed away by adding 100 μL of cold PBS to the flow-cell channels. Limited digestion was performed by adding 100 μL of trypsin, 20 μg/mL (V511, Promega, Madison, WI, United States), to the flow-cell channels for 20 min. The digested peptides were eluted from the channels with 200 μL of PBS. The eluates were centrifuged at maximum speed for one min, to remove any viable bacteria from the peptide eluate, and the supernatant was collected in a Maximum Recovery tube (MCT-150-L-C, Corning Incorporated, Reynosa, Mexico). Trypsin, 20 μg/mL (V511, Promega, Madison, WI, United States), was then added to reduce the number of missed cleavages. The peptide-trypsin suspensions were incubated overnight at 37 °C, and the enzymatic reaction was stopped by adding 40 μL of formic acid (27001-1l-M, Sigma-Aldrich, Saint Louis, MO, United States; 10% v/v) to the solution. The peptides were stored at −20 °C until further analysis was performed.

### Protein Identification by LC-MS/MS

The proteomic analysis was performed at the Proteomic Core Facility, University of Gothenburg (Sweden, www.gu.se/en/core-facilities/infrastructure-at-core-facilities/proteomics-pcf). The peptides were purified, using Pierce peptide desalting spin columns (89852, ThermoFisher Scientific, Mount Prospect, IL, United States), according to the instructions of the manufacturer, dried, and reconstituted in 15 μL of 3% acetonitrile (1.00029.1000, Merk, Darmstadt, Germany) and 0.2% formic acid (27001-1l-M, Sigma-Aldrich, Saint Louis, MO, United States). Liquid chromatography coupled with tandem mass spectrometry (LC-MS/MS) analysis was performed on an Orbitrap Exploris or an Orbitrap Fusion Tribrid mass spectrometer interfaced with an Easy-nLC1200 liquid chromatography system (ThermoFisher Scientific, Mount Prospect, IL, United States). Peptides were trapped on an Acclaim Pepmap 100 C18 trap column (100 μm × 2 cm, particle size 5 μm, ThermoFisher Scientific, Mount Prospect, IL, United States) and separated on an in-house packed analytical column (35 cm × 75 μm, particle size 3 μm, Reprosil-Pur C18, Dr. Maisch, Ammerbuch Germany). Separation was achieved using a gradient from 5% to 80% acetonitrile (1.00029.1000, Merk, Darmstadt, Germany) in 0.2% formic acid (27001-1l-M, Sigma-Aldrich, Saint Louis, MO, United States) over 90 min at a flow of 300 nL per minute. The instruments operated in data-dependent mode, where the precursor ion mass spectra were acquired at a resolution of 120,000 *m*/*z* range 380–1,500. The most intense ions, with charge states 2 to 6, were selected for fragmentation using higher-energy collisional dissociation (HCD) with collision energy settings of 30. A dynamic exclusion of 30 s was applied with a mass tolerance of 10 ppm. The isolation window was set to 1.2, and MS2 spectra were recorded at a resolution of 30,000.

### Database Searches

Raw files were analyzed using Proteome Discoverer v. 3.2.0 software (ThermoFisher Scientific, Mount Prospect, IL, United States),^49^ with Sequest HT as a search engine. The mass spectra of each triplicate were matched against their respective proteomes. Supporting Information 1 provides the corresponding accession numbers for the selected strains. The precursor mass tolerance was set to 5 ppm and the fragment mass tolerance to 0.05 Da. Tryptic peptides were accepted with one missed cleavage, variable modifications for methionine oxidation were selected, and the identified peptides were validated with Peptide-Spectrum Match (PSM) Validator.

### Label-Free Quantification Using MaxQuant

Raw files from all 11 strains were processed independently, using MaxQuant v2.4.^50^ Database searches were conducted, using the Andromeda platform search engine,^51^ within MaxQuant, against a target-decoy database. FASTA sequences for each strain were used as a reference database. Trypsin was fixed as the protease with a maximum allowance of two missed cleavages. Variable modifications were made for methionine oxidation. The mass tolerance was set to 4.5 ppm and 0.5 Da for the MS and MS/MS scans, respectively. A false discovery rate (FDR) of 1% was applied at both the peptide and the protein levels. Finally, label-free quantification (LFQ)^52^ was applied to infer quantitative information. All other parameters were kept at default settings. The abundance values were normalized according to the median intensity. Two different heatmaps were generated, one based on 462 proteins, using Euclidian distance clustering, and the other heatmap based on 77 proteins consistently detected among all strains and replicates.

### Protein Annotation Based on the Reference Proteome of the TIGR4 Strain

The proteomes of the 11 *S*. *pneumoniae* strains, analyzed in this study, were aligned with the reference proteome of the TIGR4 strain (ATCC BAA-334),^53^ using protein BLAST, to refer to the identified proteins. A 70% sequence identity and 70% coverage threshold were applied to assign protein names in the corresponding strain. The names of the proteins, their loci and UniProt entry numbers in TIGR4, were used for all the searches. The identified proteins per replicate of each of the strains were compared to determine common proteins among replicates and strains.

### Functional Annotation and Subcellular Localization

Functional annotation was performed in the OmicsBox v3. 4. 5. (BioBam, Valencia, Spain), following the Blast2GO methodology.^54^ In brief, the proteome of the TIGR4 strain was searched against multiple databases to assign functional annotations to each protein. An initial BLAST, using NCBI blast+ v. 2.13.0,^55^ was performed, limiting the search to the Bacilli (Taxon ID: 91061), followed by Gene Ontology (GO)^56,57^ term assignment with GO mapping 2025.01. InterproScan v5.72–103.0 ^58^ was used to determine protein families and domains. To identify orthologous groups, an annotation in EggNog v. 5.0.2,^59^ using the EggNog mapper v 2.1.0 option in OmicsBox, was performed. Furthermore, all the annotations were combined, redundancies were removed, and the results were filtered to obtain a final list of the annotated proteins and GO numbers and terms.

The subcellular localization of the proteins was predicted using two different databases: PsortB v3. 0. 3,^60^ and DeepLocPro v1.0.^61^ In both cases, the corresponding TIGR4 protein sequences were analyzed using their respective webtools.

### Protein Biomarker Identification through Genome and Proteome Mining of Previously Described *Strepotococcus pneumoniae* Biomarkers and Surface Antigens

To identify the protein biomarkers, two different protein BLAST searches were performed. The first BLASTp included all of the *S*. *pneumoniae* available genomes (September 2024), excluding atypical and metagenome-assembled genomes from the NCBI Genome database. A total of 8,892 genomes were retrieved and reannotated with Prokka v1.14.6,^47^ and the resulting amino acid FASTA files were used to build the database for the search with BLAST v.2.5.0. The queries for the search were 224 proteins associated with virulence, previously described by Kilian and Tettelin 2019 ^24^ to be species markers of *S*. *pneumoniae*. The search allowed for the identification of protein isoforms that shared 70% identity and aligned with 70% of the individual proteomes that were analyzed (Supporting Information 3). The second protein BLAST was done against the proteomes of the 11 strains used for surface-shaving to confirm its presence in the analyzed strains (Supporting Information 4). Mass spectrometry-identified protein accession numbers were matched against the second BLAST results for validation.

The protein biomarker list was ranked based on the number of strains harboring the biomarker protein and the number of replicates where the protein was found in the proteomic data.

To assess surface proteins previously identified as antigens,^62^ a BLAST search was performed against the genomes of the 11 strains. Proteomic data were then matched to the BLAST results, and proteins were ranked based on their detection frequencies within replicates. Additionally, *S*. *pneumoniae* antigenic proteins described in preclinical studies^63^ (mouse model) for immunization and proteins with predicted epitopes with affinity to B- and T-cells^64^ were compared to our proteomics results.

### Identification of Novel Candidate Biomarkers for *Streptococcus pneumoniae* from Proteomic Data

To identify the novel potential *S*. *pneumoniae* protein biomarkers, all identified proteins were subjected to BLAST searches against the UniProt database.^65^ Proteins that matched only those of *S*. *pneumoniae* or another species at <70% identity were selected for a second BLAST search in the NCBI database, excluding *S*. *pneumoniae* (Taxon ID: 1313). The next closest species match and the percentage similarity were considered. Additionally, the identified proteins were BLAST-searched against all *S*. *pneumoniae* genomes in the NCBI database to determine their prevalence within the species. To identify the most promising novel protein biomarkers, each protein was ranked by its prevalence in the strains and replicates (Supporting Information 5) and its abundance (Supporting Information 6). Furthermore, a cutoff value of >80% presence in the 8,892 *S*. *pneumoniae* proteomes was set; proteins showing significant similarities with non-*Streptococcus* species were excluded. Figure 1, illustrates the workflow of the data mining strategy, highlighting the pipelines, databases, and references used.

**Figure 1:**
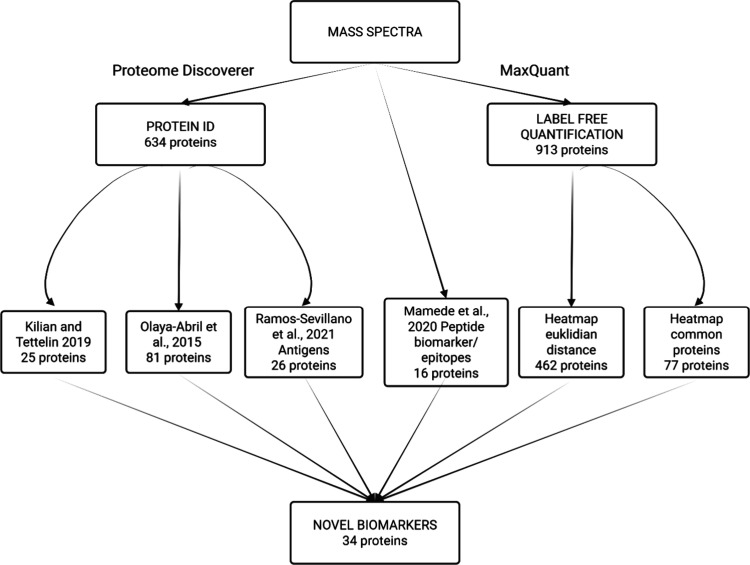
Illustration showing the data mining strategy. The raw data was processed by Proteome Discoverer and MaxQuant pipelines. Several references were used to validate the candidate biomarkers found in previous studies as well as enable discovery of novel candidates.

### Peptide Biomarker Identification

The peptide biomarkers were identified, using Microorganism Classification and Identification (MiCId), following the approach described by Alves et al., 2016.^66^ Briefly, the 33-mass spectrometry raw data files were analyzed, using a peptide database, assigning an *E*-value to each identified peptide.^67^ Prior probabilities were also calculated. The microorganism identification was selected when the *E*-values were smaller than or equal to 0.01 and the prior probability was greater than or equal to 0.01. Unique peptides were also considered for assigning peptides to their specific taxa; peptides with an *E*-value 10^–4^ were considered unique for a taxon. The top 20 peptides that were present in at least 20 matching analyses were selected as potential peptide biomarkers for *S*. *pneumoniae*. Furthermore, the unique peptides were aligned to the parent protein in the TIGR4 strain^53^ or the corresponding protein in the genome of the type strain CCUG 28588^T^. Clustal Omega^68^ was used to identify the positions in the protein where unique peptides were matching the linear sequence part of the proteins for *S*. *pneumoniae*.

## Results

### Phylogenomic Analysis Enabled the Selection of 11 Different CCUG Strains Representing *Streptococcus pneumoniae* Species Variability

To confirm the taxonomic classification of the selected genomes, ANIb and GGDC analyses values were calculated and a final data set of 300 unique pairwise values was obtained. The average ANIb and GGDC scores for all of the strains were analyzed against the *S*. *pneumoniae* type strains were determined to be 98.5% and 88.6%, respectively. The *S*. *pneumoniae* genomes showed ANIb average scores of 94.1% and 91.5%, against the type strains of *S*. *pseudopneumoniae* and *S*. *mitis*, respectively, while the corresponding GGDC average scores were 58.7% and 46.3%. The pangenome analysis showed that 1,355 proteins constituted the core genome of the 22 analyzed strains. The phylogenetic tree, based on the core genome, differentiated the strains into 11 distinct branches, representing the genomic variation of the species, from which 11 strains were selected for further proteomic analysis (Supporting Information 2).

### Lysis of Bacterial Cells Was Reduced by Optimized Surface-Shaving Protocols

The surface-shaving conditions were optimized, to minimize the presence of cytoplasmic proteins,^69^ using PBS 0.01 M as suspension buffer, trypsin (20 μg/mL) for 20 min at room temperature for digestion, followed by a second digestion overnight with trypsin (2 μg/mL) at 37 °C. These conditions yielded a higher number of surface proteins (cytoplasmic membrane, cell wall, and extracellular proteins), while reducing the number of cytoplasmic proteins detected by mass spectrometry, compared to controls and the mildest conditions tried. The proteomic results of the optimization are summarized in Supporting Information 7.

### Lipid–Protein Immobilization for Surface-Shaving Allowed the Identification of Surface Proteins Common to All the Analyzed Strains

The number of proteins identified with more than one peptide by surface-shaving and LC-MS/MS analysis ranged from 80 (strain CCUG 17113) to 482 (strain CCUG 63093). The proteome coverage achieved by surface-shaving varied among strains, from 3.7% for strain CCUG 1350, to 24.6% for strain CCUG 63093, with an average of 14%. A total of 58 proteins were commonly found in all strains, of which 18 (31%) were classified as noncytoplasmic, while the remaining 40 (69%) were identified as cytoplasmic by subcellular prediction tools (Table 1). Among the noncytoplasmic proteins, six were classified as cell wall and surface proteins (beta-N-acetylhexosaminidase, pullulanase, endo-alpha-N-acetylgalactosaminidase, endo-alpha-N-acetylglucosaminidase, beta-galactosidase, and neuraminidase A), all of which are glycan-degrading enzymes.^70^ Two proteins were located in the cytoplasmic membrane (cell division protein and general stress protein 24). Ten ribosomal proteins were identified as extracellular in one of the localization prediction programs but were classified differently by the other (Supporting Information 8).

**Table 1:** Number of Proteins Identified in *S. pneumoniae* by LPI Surface-Shaving and Tandem Mass Spectrometry^a^

Strains	Theoretical proteins	All identified proteins with >1 peptide	Proteome coverage by surface-shaving (%)	Shared proteins by replicate (%)	Common proteins in replicates	Specific proteins in the 11 strains
CCUG 1350	2,138	130	6.4	83.8	109	7
CCUG 17113	2,043	80	3.7	70.0	56	4
CCUG 28588	1,986	281	14.2	79.0	222	9
CCUG 32672*	2,024	427	21.1	94.1	402	9
CCUG 33774	1,972	244	12.4	72.1	176	1
CCUG 35180	2,032	401	19.7	81.0	315	10
CCUG 35229	1,951	249	12.8	80.3	200	1
CCUG 63093	1,956	482	24.6	86.9	419	11
CCUG 6798	1,949	172	8.8	82.7	144	1
CCUG 69380	2,201	315	14.3	80.0	252	8
CCUG 69382	2,045	317	15.5	86.4	274	4

a The experiments were performed in three biological replicates. * Only two replicates of the sample were considered for the analysis due to low quality mass spectra of the third replicate.

One replicate of strain CCUG 32672 did not yield any identified proteins; therefore, it was excluded from further analysis. The percentage of shared proteins between the three biological replicates varied between strains, ranging from 70% for strain CCUG 17113, to 94% for CCUG 32672. The results of the surface-shaved strains analyzed in triplicate are summarized in Table 1. In the cases of CCUG 1350 and CCUG 17113, a lower number of proteins were identified, compared with other strains. Unique proteins for each strain were identified, strains CCUG 6309 and CCUG 35180 exhibited the highest number of strain-unique proteins, 10 and 11 proteins, respectively.

### Surface Antigens of *Streptococcus pneumoniae* Were Identified in the Analyzed Strains

The surface antigens of *S*. *pneumoniae*, identified by Olaya-Abril et al., 2015,^62^ were used as a reference for comparison with the proteins identified in our surface-shaving experiments, detecting a total of 81 proteins (Table 2). The majority of these proteins were cell membrane proteins (60.5%), followed in prevalence by proteins from the cell wall and surface (19.7%), extracellular (13.6%), and cytoplasmic proteins (6.2%), according to the subcellular prediction tool DeepLocPro v. 1.0.^61^ Figure 2 represent schematically the location of the identified antigenic protein from the pneumococcal surface. The 25 most frequently detected proteins were present in at least seven of the different surface-shaved analyzed strains and in at least 18 replicates (Table 2). It is worth mentioning that the proportion of proteins located in the cytoplasmic membrane (ten), cell wall and surface (seven), extracellular (two) and cytoplasmic (one) was the same as in the set of 81 proteins. Six proteins, beta-galactosidase, exo-alpha-sialidase, ATP-dependent zinc metalloprotease FtsH, sialidase A, endo-alpha-N-acetylglucosaminidase and beta-N-acetylhexosaminidase, were present in all replicates of the analyzed strains. These proteins were predominantly localized to the cell walls of *S*. *pneumoniae*, with the exception of the ATP-dependent zinc metalloprotease FtsH, which is located in the cytoplasmic membrane, according to prediction tools.

**Table 2:** Protein Ranking of the Surface Antigens Found by Surface-Shaving in the 11 Analyzed Strains^a^

Rank	Antigens	Gene name	Locus in TIGR4 strain	UniProt entry number	Protein description	No. of strains with the protein	MS runs with the peptide	*In-silico* protein detection (%)	Subcellular localization
1	B2IMV9	*bgaA*	SP_0648	A0A0H2UP19	Beta-galactosidase	11	32	100	Cell wall and surface
2	C1C8T0		-	C1C8T0	Exo-alpha-sialidase	11	32	100	Cell wall and surface
3	P59652	*ftsH*	SP_0013	O69076	ATP-dependent zinc metalloprotease FtsH	11	32	100	Cytoplasmic membrane
4	P62576	*nanA*	-	P62576	Sialidase A	11	32	100	Cell wall and surface
5	Q2MGH6	*SpGH101*	SP_0368	Q2MGH6	Endo-alpha-N-acetylgalactosaminidase	11	32	100	Cell wall and surface
6	Q8CZ52	*engase*	SP_0498	A0A0H2UNT5	Putative endo-beta-N-acetylglucosaminidase	11	32	100	Cell wall and surface
7	Q8DRL6	*strH*	SP_0057	P49610	Beta-N-acetylhexosaminidase	11	32	100	Cell wall and surface
8	Q8DRA6	*spuA*	SP_0268	A0A0H2UNG0	Pullulanase (alpha-dextrin endo-1,6-alpha-glucosidase)	10	29	100	Cell wall and surface
9	Q8DQC2		SP_0845	A0A0H2UPF3	Lipoprotein	10	28	100	Unknown^b^
									Cytoplasmic membrane^c^
10	Q8CYJ8	*mltG*	SP_1518	A0A0H2UQS8	Endolytic murein transglycosylase	10	27	100	Unknown^b^
									Cytoplasmic membrane^c^
11	Q8DMY4	*pcsB*, *usp45*	SP_2216	A0A0H2US70	Peptidoglycan hydrolase PscB	10	27	100	Extracellular
12	Q8DN07	*glpO*, *exp6*	SP_2185	P35596	Alpha-glycerophosphate oxidase	9	27	100	Cytoplasmic membrane
13	Q8DN58	*cap4C*	SP_2092	P58313	UTP-glucose-1-phosphate uridylyltransferase	10	27	100	Cytoplasmic
14	Q8DQI1	*livJ*	SP_0749	A0A0H2UPD6	Branched-chain amino acid ABC transporter	9	25	100	Unknown^b^
					Amino acid-binding protein				Cytoplasmic membrane^c^
15	P59214	*malX*	SP_2108	P59213	Maltooligosaccharide ABC transporter solute binding lipoprotein	8	23	100	Unknown^b^
									Cytoplasmic membrane^c^
16	Q8DNE1	*yidC1*	SP_1975	Q97NP5	Membrane protein insertase YidC1	8	23	100	Cytoplasmic membrane
17	Q8DQF9		SP_0785	A0A0H2UPI5	RND efflux pump membrane fusion protein	8	23	100	Unknown^b^
					Barrel-sandwich domain containing protein				Cytoplasmic membrane^c^
18	C1CIL8	*lacE-2*	SP_1185	A0A0H2UQ49	PTS system. lactose-specific IIBC components	8	22	100	Cytoplasmic membrane
19	Q8DPY8		SP_1027	A0A0H2UPZ4	Extracellular protein	8	22	100	Unknown^b^
									Extracellular^c^
20	Q8DQ24	*prsA*	SP_0981	Q97R51	Foldase protein prsA	8	22	100	Cytoplasmic membrane
21	P67283	*rny*	SP_1739	P67282	Ribonuclease Y	8	20	100	Cytoplasmic^b^
									Cytoplasmic membrane^c^
22	Q59947	*iga*	SP_1154	Q97QP7	Immunoglobulin A1 protease	7	20	90.9	Cell wall and surface
23	B2IMV5		SP_0641	A0A0H2UP55	Serine protease	7	19	100	Cell wall and surface^b^
					Subtilase family				Extracellular^c^
24	Q8DR55	*mapZ*	SP_0374	A0A0H2UNG3	Midcell-anchored protein Z	7	19	100	Unknown^b^
									Cytoplasmic membrane^c^
25	Q8DPA5		SP_1410	A0A0H2UQV1	Exported hydrophilic protein	7	18	100	Unknown^b^
									Cytoplasmic membrane^c^
26	Q8DQT0	*pnp*	SP_0588	Q97S28	Polyribonucleotide nucleotidyltransferase	8	18	100	Cytoplasmic
27	Q8DR59	*ponA*, *PBP-1A*	SP_0369	Q04707	Penicillin-binding protein 1A, DD-transpeptidase	7	18	100	Extracellular
28	Q8DP07	*lytC*	SP_1573	A0A0H2XFA2	Lysozyme	7	17	81.8	Unknown^b^
									Extracellular^c^
29	Q8CYC9		SP_1833	A0A0H2URN9	Cell wall surface anchor family protein	6	15	100	Cell wall and surface
30	Q8DQ95		SP_0877	A0A0H2UPH7	PTS system. fructose specific IIABC components	6	13	100	Cytoplasmic membrane
31	Q8DQG5		SP_0771	A0A0H2UPH6	Peptidyl-prolyl cis–trans isomerase	5	12	100	Unknown^b^
									Extracellular^c^
32	Q8DNT2		SP_1716	A0A0H2UR60	ABC-2 type transporter transmembrane domain-containing protein	5	11	100	Cytoplasmic membrane
33	Q8CWR4		SP_1174	A0A0H2UQ39	Conserved domain protein	4	10	100	Unknown^b^
									Cell wall and surface^c^
34	Q8DNS0	*stkP*	SP_1732	Q97PA9	Serine/threonine protein kinase StkP	5	10	100	Cytoplasmic membrane
35	Q8DRB1		SP_0263	Q97SR2	Putative zinc metalloprotease	4	10	100	Cytoplasmic membrane
36	Q8CWT8		-	Q8CWT8	UDP-glucose 6-dehydrogenase	3	9	36.4	Cytoplasmic membrane^b^
									Cytoplasmic^c^
37	Q8DNI1	*amiA*	SP_1891	P18791	Oligopeptide-binding protein AmiA	3	9	90.9	Cell wall^b^
									Cytoplasmic membrane^c^
38	Q8DPM3		SP_1218	Q8DPM3	Sortase (LPXTG specific)	4	9	100	Unknown^b^
									Cytoplasmic membrane^c^
39	Q8DRI0	*pspA*	-	Q8DRI0	Surface protein pspA	3	9	90.9	Extracellular^b^
									Cell wall and surface^c^
40	Q8DP20		SP_1551	A0A0H2UQU8	Cation-transporting ATPase. E1-E2 family	3	8	100	Cytoplasmic membrane
41	Q8DPD6	*MsrR*	SP_1368	A0A0H2UQH2	Regulatory protein MsrR	3	8	100	Cytoplasmic membrane
42	Q8DQE5	*ezrA*	SP_0807	Q97RK0	Septation ring formation regulator EzrA	4	8	100	Cytoplasmic^b^
									Cytoplasmic membrane^c^
43	Q8DQN5	*zmpB*	-	Q8DQN5	Zinc metalloprotease ZmpB	3	8	36.4	Cell wall and surface
44	Q8DNE7		SP_1967	A0A0H2URQ4	Endopeptidase La	3	7	100	Unknown^b^
									Cytoplasmic membrane^c^
45	Q8DNS8		SP_1722	A0A0H2UR65	PTS system IIABC components	3	7	72.7	Cytoplasmic membrane
46	Q8DR99		SP_0282	A0A0H2UNG8	PTS system. mannose-specific IID component	3	7	100	Cytoplasmic membrane
47	P0A4G1	*aliB*	SP_1527	P0A4G0	Oligopeptide-binding protein AliB	2	6	100	Cell wall^b^
									Cytoplasmic membrane^c^
48	Q8CYB3		SP_1926	A0A0H2URJ8	DUF4231 domain-containing protein	2	6	100	Cytoplasmic membrane
49	Q8DN05	*cbpA*	SP_2190	A0A0H2US50	Choline binding protein A	2	6	100	Extracellular^b^
									Cell wall and surface^c^
50	Q8DN88	*comGC*, *cglC*	SP_2051	A0A0H2URU9	Competence protein ComGC	2	6	100	Unknown^b^
									Cytoplasmic membrane^c^
51	Q8DR61	*aliA*, *exp1*, *plpA*	SP_0366	P35592	Oligopeptide-binding protein AliA	2	6	81.8	Cell wall^b^
									Cytoplasmic membrane^c^
52	Q97NB5	*pcpA*	SP_2136	A0A0H2USF9	Choline binding protein PcpA	2	6	100	Unknown^b^
									Extracellular^c^
53	P64167	*ftsK*	SP_0878	P64166	DNA translocase FtsK	2	5	100	Cytoplasmic membrane
54	Q8CY83		SP_2057	A0A0H2URY7	Acyltranferase 3 domain-containing protein	2	5	100	Cytoplasmic membrane
55	Q8CYI8		-	Q8CYI8	G5 domain containing protein	2	5	27.3	Cell wall and surface
56	Q8DP15		SP_1560	A0A0H2UQV5	Lipoprotein	2	5	100	Cytoplasmic membrane
57	Q8DPB7		SP_1394	A0A0H2UQR1	Amino acid ABC transporter	2	5	90.9	Unknown^b^
					Amino acid-binding protein				Cytoplasmic membrane^c^
58	Q8DRJ2		SP_0103	A0A0H2UMZ0	Putative capsular polysaccharide biosynthesis protein	2	5	100	Cytoplasmic membrane
59	P0A399	*dltA*	SP_2176	P0A398	d-Alanine-d-alanyl carrier protein ligase	3	4	100	Cytoplasmic
60	Q8DPH4	*lemA*	SP_1284	A0A0H2UQI3	lemA protein	2	4	100	Unknown^b^
									Cytoplasmic membrane^c^
61	Q8DPK5		SP_1241	A0A0H2UQ91	Amino acid ABC transporter. Amino acid-binding protein/permease protein	2	4	100	Cytoplasmic membrane
62	Q8DQ87	*pepX*	SP_0894	Q97RC8	Xaa-pro dipeptidyl-peptidase	2	4	100	Extracellular^b^
									Cytoplasm^c^
63	C1CRC0		SP_0462	A0A0H2UNT6	Cell wall surface anchor family protein	1	3	18.2	Unknown^b^
									Cell wall and surface^c^
64	P59206	*lytB*	SP_0965	P59205	Endo-beta-N-acetylglucosaminidase	1	3	63.6	Extracellular
65	P59676	*pbpX*	SP_0336	P14677	Penicillin-binding protein 2X	1	3	100	Cytoplasmic membrane
66	Q8CY68		SP_2132	A0A0H2US28	Band 7 domain-containig protein	2	3	100	Cytoplasmic membrane
67	Q8CYX1		SP_0899	A0A0H2UPK1	Lipoprotein	1	3	100	Unknown^b^
									Cytoplasmic membrane^c^
68	Q8CZ16		SP_0667	A0A0H2UP31	Putative pneumococcal surface protein	2	3	100	Unknown^b^
									Extracellular^c^
69	Q8DQ07	*PhtE*	SP_1004	A0A0H2UPR3	Pneumococcal histidine triad protein E	1	3	100	Unknown^b^
									Cytoplasmic membrane^c^
70	Q8DQH3	*ftsX*	SP_0757	A0A0H2UP94	Cell division protein FtsX	1	3	100	Cytoplasmic membrane
71	Q97NF0		SP_2093	A0A0H2US03	Uncharacterized protein	1	3	18.2	Unknown^b^
									Extracellular^c^
72	P0A3S4	*endA*	SP_1964	P0A3S3	DNA-entry nuclease	1	2	100	Cytoplasmic membrane
73	Q8CWU2	*cps4A*	SP_0346	A0A0H2UNF1	Capsular polysaccharide biosynthesis protein Cps4A	1	2	81.8	Cytoplasmic membrane
74	Q8CWU3		SP_0314	Q54873	Hyaluronate lyase	1	2	90.9	Cell wall and surface
75	Q8DN70		SP_2075	A0A0H2USC5	ABC transporter. ATP-binding/permease protein	1	2	100	Cytoplasmic membrane
76	Q8DN72		SP_2073	A0A0H2US37	ABC transporter. ATP-binding/permease protein	1	2	100	Cytoplasmic membrane
77	Q8DNB6	*pbp2A*	SP_2010	A0A0H2URT5	Peptidoglycan glycosyl transferase	1	2	100	Cytoplasmic membrane
78	Q8DP34		-	Q8DP34	ABC transporter truncation	1	2	81.8	Cytoplasmic membrane
79	Q8DPQ2		SP_1175	A0A0H2UQ89	Conserved domain protein	1	2	90.9	Unknown^b^
									Cytoplasmic membrane^c^
80	Q8DQ10		SP_1000	A0A0H2UPR5	Thioredoxin family protein	1	2	100	Cell wall^b^
									Cytoplasmic membrane^c^
81	Q8DQ62	*cbpE*	SP_0930	A0A0H2UPM5	Choline binding protein E	1	1	90.9	Extracellular

a The antigens described in Olaya-Abril et al., 2015,^62^ were retrieved from UniProt database.^65^ A BLAST search based on the 70% coverage and 70% identity was performed to identify the proteins found by surface-shaving. The proteins were ranked according to the number of replicates and strains that presented the protein. Proteins without a match in the TIGR4 ^54^ proteome are referred in the table with the same accession number as described elsewhere and lack an SP number. The subcellular localization of the proteins was predicted with two different databases,^60,61^

b PsortB.

c DeepLocPro.

**Figure 2:**
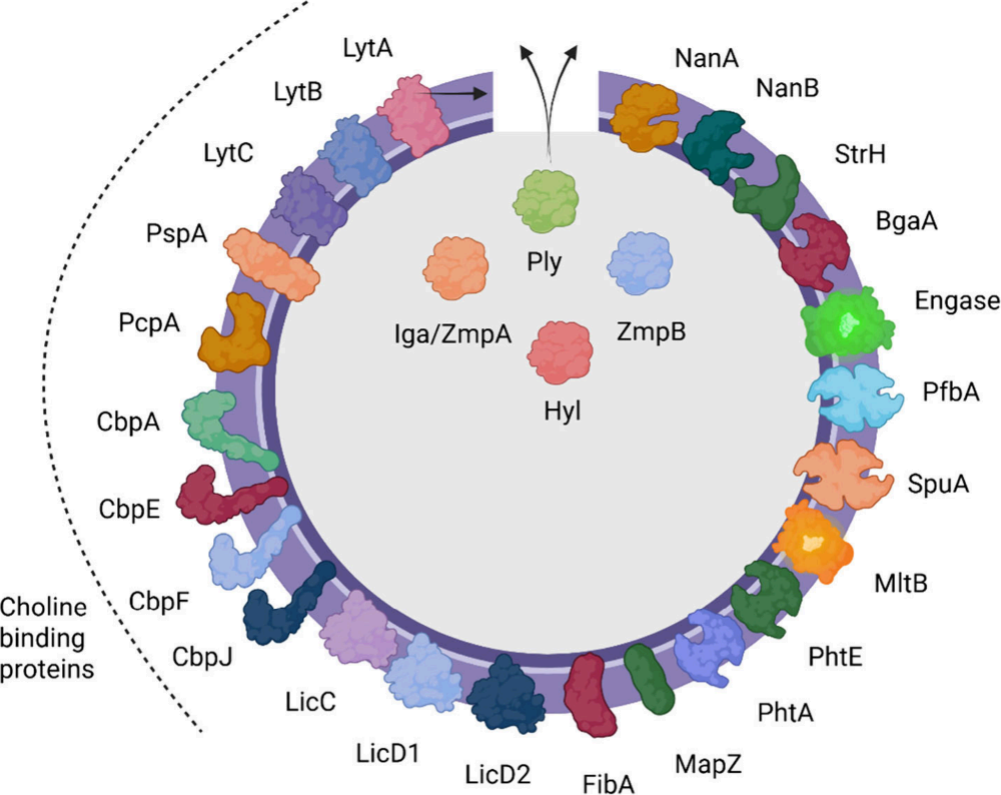
*Streptococcus pneumoniae* schematic localization of the principal protein biomarkers found by surface-shaving. The illustrated protein virulence factors and choline binding proteins are represented according to their respective subcellular localization. Figure created with BioRender.

After comparing the identified proteins to the list described by Ramos-Sevillano et al. 2021,^63^ where proteins with antigenic properties in mice were highlighted, it was possible to confirm that the lipoproteins, such as ABC transporter, substrate binding protein (SP_0148), lipoproteins (SP_0149, SP_0845), oligopeptide-binding proteins AliB (SP_1527) and AmiA (SP_1891), and foldase protein PrsA (SP_0981), were part of our identified proteins (data not shown). Furthermore, the LPXTG motif proteins beta-N-acetylhexosaminiadase (SP_0057), endo-alpha-N-acetylgalactosaminidase (SP_0368), and serine protease PrtA (SP_0641) ranked high in our protein lists, as well as the nonclassical surface proteins, such as enolase (SP_1128), serin protease (SP_2239) and glycelraldehyde-3-phosphate dehydrogenase (SP_2012).

### Previously Identified Unique Proteins for *Streptococcus pneumoniae* Were Experimentally Verified in the Analyzed Strains

Twenty-five proteins out of the 224 unique biomarkers of *S*. *pneumoniae* described by Kilian and Tettelin, 2019 ^24^ were identified in the surface-shaving experiments, thereby confirming their presence in the analyzed strains. Among the most frequently detected proteins, ornithine carbamoyltransferase (P65607), arginine deaminase (Q97NA4), pneumolysin (P0C2J9) and carbamate kinase (A0A0H2US27) were found in eight and seven strains, respectively. All unique proteins were ranked according to their presence in the strains and their replicates, as described in Table 3. The subcellular localization analysis indicated that 56% of the unique proteins were noncytoplasmatic. Specifically, six proteins were localized in the cytoplasmic membrane, five proteins in the cell wall and surface, and three proteins in the extracellular space. Notably, six proteins (cell wall surface anchor protein family, DUF4231 domain-containing protein, choline binding protein PcpA, choline binding protein A, lipoprotein, and hyaluronate lyase) were shared between the two protein lists presented in this study (Tables 2 and 3).

**Table 3:** Rank of the Unique Protein Biomarkers for *S. pneumoniae* Confirmed by LPI Surface-Shaving and Tandem Mass Spectrometry^a^

							*In-silico* presence of the protein		
Rank	Locus in TIGR4 strain	Gene name	UniProt entry number	Protein description	No. of strains with the protein	MS runs with the protein	11 strains (%)	***S***. pneumoniae NCBI strains (%)	Subcellular localization
1	SP_2150	*arcB*	P65607	Ornithine carbamoyltransferase	8	21	100	96.1	Cytoplasmic
2	SP_2148	*arcA*	Q97NA4	Arginine deiminase	8	20	90.9	95.3	Cytoplasmic
3	SP_1923	*ply*	P0C2J9	Pneumolysin	7	19	100	98.7	Extracellular
4	SP_2151	*arcC*	A0A0H2US27	Carbamate kinase	7	19	100	96.1	Cytoplasmic
5	SP_1833		A0A0H2URN9	Cell wall surface anchor family protein	6	15	100	97.0	Cell wall and surface
6	SP_0860	*pcp1*	P65678	Pyrrolidone-carboxylate peptidase	5	14	100	99.8	Cytoplasmic
7	SP_0886		A0A0H2UPJ1	Site-specific DNA-methyltransferase (adenine-specific)	5	13	81.8	95.1	Unknown^b^
									Cytoplasmic^c^
8	SP_1926		A0A0H2URJ8	DUF4231 domain-containing protein	2	6	90.9	99.0	Unknown^b^
									Cytoplasmic membrane^c^
9	SP_2136	*pcpA*	A0A0H2USF9	Choline binding protein PcpA	2	6	63.6	54.0	Unknown^b^
									Extracellular^c^
10	SP_2190	*cbpA*	A0A0H2US50	Choline binding protein A	2	6	18.2	32.6	Extracellular^b^
									Cell wall and surface^c^
11	SP_0510	*hsdR*	A0A0H2UNV7	Type I restriction-modification system	2	6	81.8	97.3	Cytoplasmic
				R subunit					
12	SP_1937	*lytA*	P06653	Autolysin	2	6	100	90.2	Extracellular
13	SP_0578	*bglA-2*	A0A0H2UP35	6-Phospho-beta-glucosidase	2	5	100	98.9	Cytoplasmic
14	SP_0251		A0A0H2UN81	Putative formate acetyltransferase	3	5	100	99.8	Cytoplasmic
15	SP_1884		A0A0H2URK8	Trehalose PTS system	2	5	81.8	99.4	Cytoplasmic membrane
				II ABC component					
16	SP_0117	*pspA*	A0A0H2UMZ8	Pneumococcal surface protein A	1	3	9.1	10.8	Extracellular^b^
									Cell wall and surface^c^
17	SP_0899		A0A0H2UPK1	Lipoprotein	1	3	100	99.8	Unknown^b^
									Cytoplasmic membrane^c^
18	SP_2153		A0A0H2US39	Peptidase M20/M25/M40 family	1	3	100	95.1	Cytoplasmic
19	SP_2084	*pstS2*	P0C2M5	Phosphate-binding protein PstS 2	1	3	100	99.8	Unknown^b^
									Cytoplasmic membrane^c^
20	SP_0082		A0A0H2UN40	Cell wall surface anchor family protein	1	2	45.5	5.5	Cell wall and surface
21	SP_0314		Q54873	Hyaluronate lyase	1	2	90.9	95.8	Cell wall and surface
22	SP_1500	*aatB*	A0A0H2UQY7	Amino acid ABC transporter	1	2	100	97.8	Unknown^b^
				Amino acid binding protein					Cytoplasmic membrane^c^
23	SP_2087	*pstB3*	P0A2V8	Phosphate import ATP-binding protein PstB 3	1	2	100	99.9	Cytoplasmic membrane
24	SP_0950		A0A0H2UPN4	Acetyltransferase	1	2	100	84.8	Cytoplasmic
				GNAT family					
25	SP_1894	*gtfA*	A0A0H2URT4	Sucrose phosphorylase	1	1	100	98.9	Cytoplasmic

a The unique protein biomarkers described in Kilian and Tettelin, 2019,^24^ for *S*. *pneumoniae* were confirmed experimentally by surface-shaving and tandem mass spectrometry. A BLAST search based on the 70% coverage and 70% identity was performed against the TIGR4 strain^53^ to match the identity of the proteins found by surface-shaving. The subcellular localization of the proteins was predicted with two different databases.^60,61^

b PsortB.

c DeepLocPro. The proteins were ranked according to the number of replicates and strains that presented the protein.

### In-Depth Proteome Mining Elucidated the Prevalence of the *Streptococcus pneumoniae* Biomarkers

The proteomic data from the analyzed strains (913 proteins) were used to identify novel biomarkers for *S*. *pneumoniae*. An initial list with 91 proteins was obtained after the BLAST analysis against the UniProt database.^65^ The protein list was reduced to 73 proteins after filtering out species not included in the genus *Streptococcus*, based on NCBI BLAST^55^ results. Of those, 41 were also detected in *S*. *pseudopneumoniae*, 16 proteins were shared with *S*. *mitis* and seven with *Streptococcus oralis*. Moreover, 42 out of the 73 proteins were present in more than 80% of all of the available *S*. *pneumoniae* proteomes. The proteins were ranked according to their presence in the *S*. *pneumoniae* proteomes. Notably, 34 of the listed biomarkers are considered to be novel candidate biomarkers for *S*. *pneumoniae*. The 25 top-ranked proteins, including endo-beta-N-acetylglucosaminidase, pneumolysin, multiligand-binding adhesin PfbA, uncharacterized protein, and serine protease, are visualized in a Whisker′s plot (Figure 3 and Supporting Information 9). Interestingly, nine proteins of the 91 matched to other Gram-positive and Gram-negative species, with a high prevalence among *S*. *pneumoniae*. Furthermore, proteins with lower similarities to other species were often exclusive to only a few *S*. *pneumoniae* proteomes.

**Figure 3:**
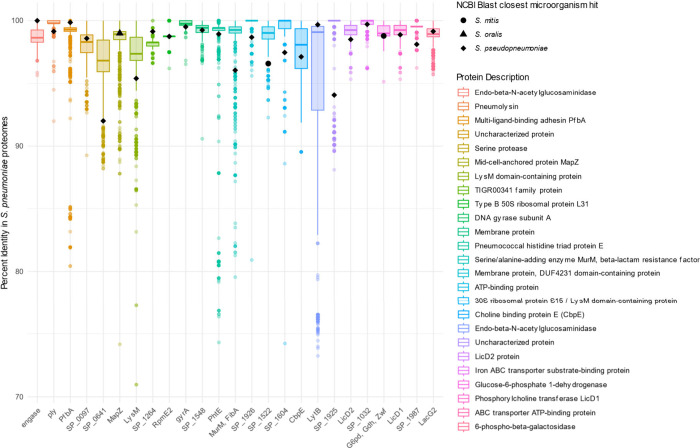
Top 25 candidate protein biomarkers identified from the proteomic analysis are shown as color-coded whisker plots with corresponding BLAST annotations. The biomarkers were selected based on the following criteria: (1) high label-free quantification (LFQ) intensity observed in ≥ 80% of replicates across the 11 proteomic data sets analyzed; (2) consistent presence among the 8,992 proteomes identified; and (3) high sequence similarity to *Streptococcus* species, as determined by NCBI BLAST. The *Y*-axis represents percent identity in *S. pneumoniae* proteomes (protein accession number used for BLAST analysis are listed in Supporting Information 9), while the *X*-axis displays protein names and their corresponding BLAST IDs. Colored whiskers represent the top 25 candidate biomarkers, and the closest BLAST hits ID to other *Streptococcus* species (*S. mitis*, *S. oralis*, and *S. pseudopneumoniae*) are shown in black.

### Protein Relative Abundance Level Profiling Demonstrates Similarities in Detected Surfaceome between the 11 Analyzed Strains

To identify candidate protein biomarkers with high and consistent abundance across multiple strains, protein relative abundance levels were estimated by MaxQuant LFQ intensities^50–52^ (Supporting Information 6). The results were visualized by generating heatmaps in Figure 4 and Supporting Information 10. The proteins that were consistently detected across all strains and replicates (77 proteins; Figure 4) showed different abundance patterns for the different strains. Among these were ribosomal proteins; structural proteins; and also relevant virulence factors such as beta-N-acetylhexosaminidase, pullulanase, endo-alpha-N-acetylgalactosaminidase, endo-beta-N-acetylglucosaminidase, beta-galactosidase and Sialidase A (neuraminidase A); and “moonlighting”^71^ proteins, such as elongation factor G, molecular chaperone DnaK, HU family DNA-binding protein, enolase, elongation factor Tu, transcription elongation factor GreA and Glyceraldehyde-3-phosphate dehydrogenase (GAPDH) were differentiated. This heatmap (Figure 4) highlights proteins that could be interesting targets for diagnostics and treatment, including many surface-associated virulence factors and potential new targets. In Supporting Information 10, the heatmap, based on 462 proteins, displays similarities in protein relative abundance for the analyzed strains. The strains clustered together also showed high reproducibility within replicates. However, CCUG 69380 and CCUG 69382 were indistinguishable. The strains CCUG 32672, CCUG 63093, and CCUG 35180 displayed a higher number of proteins. To highlight the similarities in abundances of interesting candidate biomarker proteins shared between strains, a ranked list based on the protein abundances of the type strain CCUG 28588 is included in Supporting Information 11. Moreover, the table displays representative MS/MS spectra of the identified peptides corresponding to four of the candidate proteins, all showing high abundances among all 11 strains and triplicate analyses.

**Figure 4:**
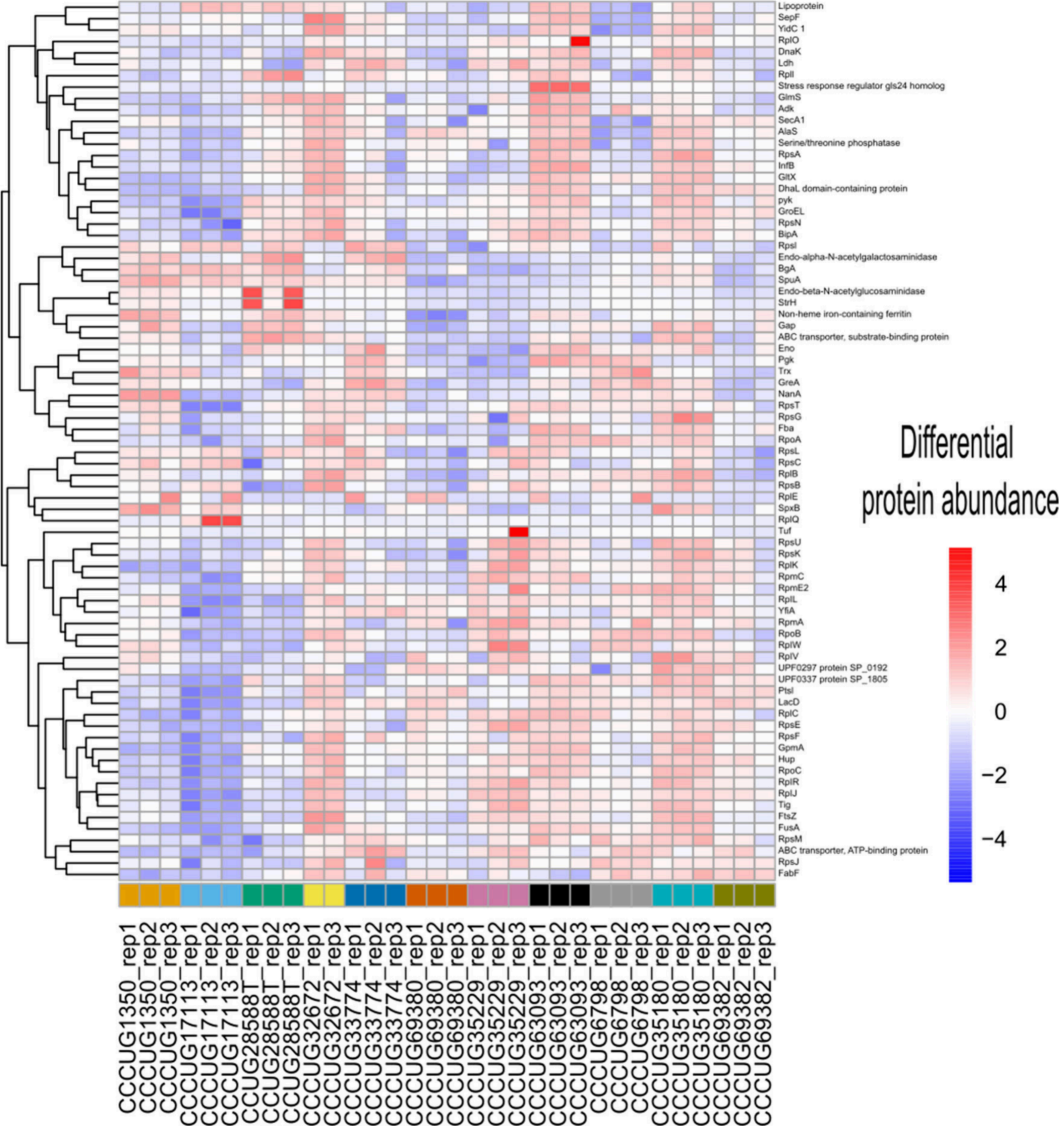
Protein abundances of the 77 common proteins found in the 11 *S. pneumoniae* strains. Heatmap and dendrogram based on Euclidian distance showing similar protein relative abundance patterns among the three biological replicates.

### Surface Proteins Contain Unique Peptides That Allow the Identification of *Streptococcus pneumoniae* as Determined by MiCid Pipeline

The 33 raw mass spectrometry files were analyzed through the MiCId pipeline^66^ in order to identify unique peptides for *S*. *pneumoniae*. A total of 5,611 peptides were detected, of which 23% (1,279 peptides) were unique to the *Streptococcus* genus and 19% (1,076 peptides) were specific to *S*. *pneumoniae*. Table 4 summarizes the top 20 peptide sequences that were present in at least 66% of all the mass spectra files with high-quality spectra. Peptide lengths ranged from 10 to 21 amino acids. Interestingly, two peptides belonging to two different proteins, beta-galactosidase and stress response regulator gls24 homologue, were found in all analyzed strains.

**Table 4:** Rank of the Unique Peptide Biomarkers for *S. pneumoniae* Identified by LPI Surface-Shaving and Tandem Mass Spectrometry According to the MiCId Database^a^

Rank	UniProt entry number	Locus in TIGR4 strain	Gene name	Description protein of origin	Subcellular localization	Unique peptide sequence	MS runs with the peptide	Peptide length (AA)	Amino acid position in the parent protein
1	A0A0H2UP19	SP_0648	*bgaA*	Beta-galactosidase	Cell wall and surface	TEQ­SEP­SST­EAI­ASEK	32	16	80–96
2	A0A0H2URD5	SP_1804		Stress response regulator gls24 homologue	Cytoplasmic^b^	VSD­VAE­STG­EFT­SEQ­FEK	32	18	145–163
					Cytoplasmic membrane^c^				
3	*Q54875	-	*iga*	Immunoglobulin A1 protease	Cell wall and surface	TTS­DFE­VSN­QEK	30	12	193–205
4	*P62575	-	*nanA*	Sialidase A	Cell wall and surface	SQP­SSE­TELS­GNK	28	13	42–55
5	P0CB77	SP_1664	*sepF*	Cell division protein SepF	Cytoplasmic	QQE­LAN­QSQR	28	10	66–76
6	Q2MGH6	SP_0368	*SpGH101*	Endo-alpha-N-acetylgalactosaminidase	Cell wall and surface	VGE­DQG­SPE­VTD­GPK	27	15	72–87
7	*P62575	-	*nanA*	Sialidase A	Cell wall and surface	SQP­SSE­TEL­SGN­KQE­QER	25	18	42–60
8	A0A0H2UNG0	SP_0268	*spuA*	Pullulanase A	Cell wall and surface	NTT­TLA­QPL­TDT­AAG­SGK	25	18	57–75
9	Q97QP7	SP_1154	*iga*	Immunoglobulin A1 protease	Cell wall and surface	QSS­DSQ­EQL­AEHK	25	13	268–280
10	P0A2W0	SP_0418	*acpP*	Acyl carrier protein	Cytoplasmic	TVG­DLV­AYV­EEQ­AK	24	14	60–74
11	A0A0H2UP19	SP_0648	*bgaA*	Beta-galactosidase	Cell wall and surface	IAP­NTD­LNS­VDK	24	12	1068–1080
12	A0A0H2UQJ0	SP_1269	*pck*	Choline kinase	Cell wall and surface	GLA­SYG­GSDEK	24	11	251–262
13	A0A0H2UNT5	SP_0498	*engase*	Putative endo-beta-N-acetylglucosaminidase	Cell wall and surface	ASE­VVA­ETP­SAE­AKPK	23	16	115–131
14	Q54970	SP_0730		Pyruvate oxidase	Cytoplamic membrane^b^	HAD­QDA­IYS­IDV­GN­TTQ­TSTR	23	21	376–397
					Cytoplasmic^c^				
15	*P62575	-	*nanA*	Sialidase A	Cell wall and surface	EDV­ETN­ASN­GQR	22	12	86–98
16	P49610	SP_0057	*strH*	Beta-N-acetylhexosaminidase	Cell wall and surface	AQQ­DTID­QAIAK	22	12	590–602
17	A0A0H2URD5	SP_1804		Stress response regulator gls24 homologue	Cytoplamic^b^	GAA­NGV­VSH­ENTR	22	13	187–200
					Cytoplasmic membrane^c^				
18	Q97RN9	SP_0762	*metK*	S-Adenosylmethionine synthetase	Cytoplasmic	IDT­FGT­GTV­AES­QLEK	21	16	324–340
19	P35594	SP_0820	*clpE*, *exp4*	ATP-dependent Clp protease ATP-binding subunit ClpE	Cytoplamic membrane^b^	ITD­QDTvPII­SEK	21	12	404–416
					Cytoplasmic^c^				
20	Q97R82	SP_0945	*frr*	Ribosome recycling factor	Cytoplasmic	ALN­ASD­IGIT­PAN­DGS­VIR	21	19	81–100

a The peptide sequences were aligned to the parent protein in the TIGR4 proteome. Proteins marked with * did not match the TIGR4 protein sequence therefore were aligned to the corresponding type strain protein sequence. The subcellular localization of the proteins was predicted with two different databases.

b PsortB.

c DeepLocPro. The peptides were ranked according to the number of replicates that had the peptide. AA, amino acids.

The unique peptide sequences are for *S*. *pneumoniae* species belonged to 16 different parent proteins, 12 of which possessed only one of these peptide sequences. Beta-galactosidase, stress response regulator gls24, and immunoglobulin A1 protease have two unique peptide sequences that, according to the MiCId database,^66^ identify *S*. *pneumoniae*; the peptides were located on the exterior regions of the 3D protein structure (Figure 5).

**Figure 5:**
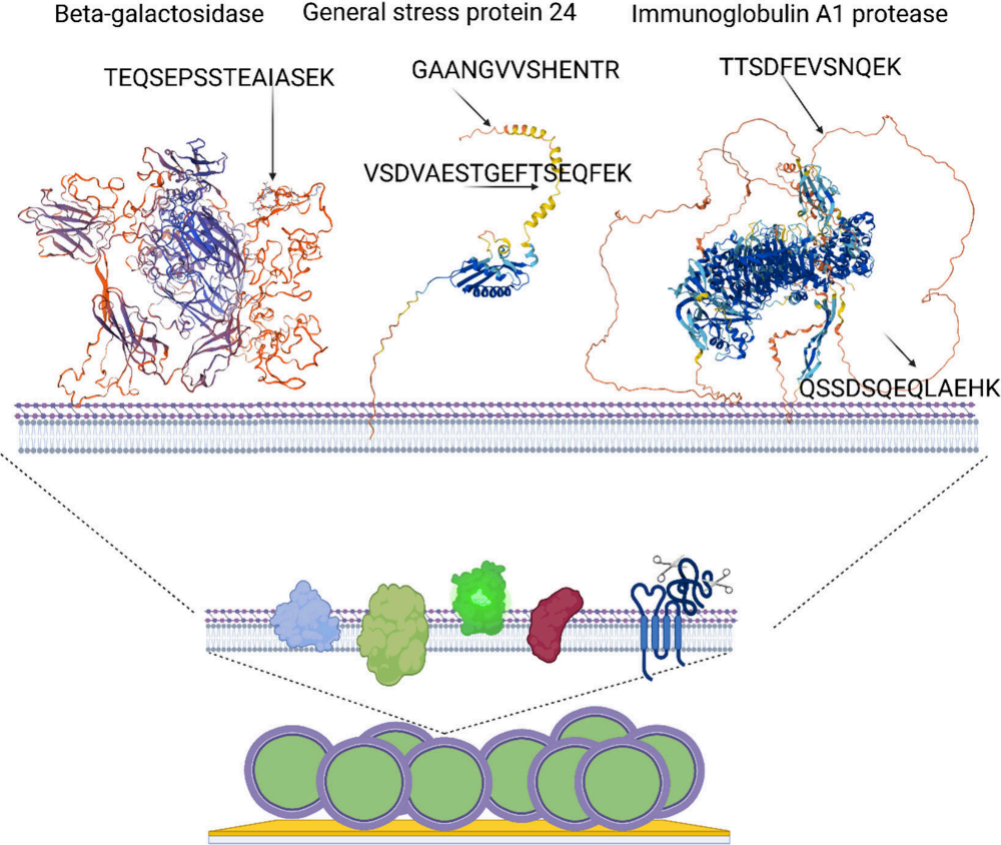
Generation of species of unique peptides from surface proteins. Schematic representation of the surface-shaving process in detail for three surface proteins with various unique peptide biomarkers. In the top drawing from left to right, beta-galactosidase, general stress protein 24 and immunoglobulin A1 protease are represented. The location of the species-unique peptides in the protein structure is indicated by arrows. Figure created with BioRender.

Meanwhile, sialidase A presented three different unique peptide sequences. Two of these sequences were aligned at residue S40 of the protein P62575, with 13 and 18 amino acids, respectively, and sharing most of their sequence, except for the last three amino acids. Three of the unique peptides matched proteins from the *S*. *pneumoniae* biomarker list reported by Kilian and Tettelin, 2019,^24^ cell division protein SepF (P0CB77), immunoglobulin A1 protease (Q97QP7), and putative endo-beta-N-acetylglucosaminidase (A0A0H2UNT5). The majority of proteins are from which *S*. *pneumoniae* unique peptides originated were noncytoplasmic, with seven different proteins localized in the cell wall and surface. These cell wall proteins contained 11 of the unique peptides, whereas five peptides originated from cytoplasmic proteins (Table 4). The subcellular localization of three proteins, stress response regulator gls24 homologue, pyruvate oxidase, and ATP-dependent Clp protease ATP-binding subunit ClpE, was not possible to determine by the databases used for localization assignment.

Additionally, all the identified peptides were compared to a list of previously described promising epitopes for B- and T-cell recognition.^64^ Two peptide sequences, NDG­AVA­LAR (histidine triad protein) and AVT­VDE­YQK (Oligopeptide ABC transporter), were identified in the proteomic data from strains CCUG 32672 and CCUG 28588, respectively.

## Discussion

In this study, we experimentally identified the presence of candidate protein and peptide biomarkers of *S*. *pneumoniae*, by using surface-shaving and tandem mass spectrometry. We focused on the surface proteins as species-unique biomarkers compared to previous studies describing whole-cell-lysates,^26^ to enable the discovery of surface-accessible biomarker proteins and peptides.^41^ Targeting the surfaceome facilitates the identification of biomarkers^35,39,41^ that are suitable for downstream detection using nonmass spectrometry-based detection methods, e.g., antibody- and sensor-based approaches.^62^ Surfaceomics also enables the discovery of novel therapeutic targets for treatment, including e.g., protein-based vaccines and therapeutic antibodies.^18,38,43,62,64^ This study was part of a Marie Skłodowska-Curie Innovation Training Network (ITN), funded by the European Commission, Horizon 2020 Program, where the end-goal of the project was the generation of graphene-based sensors for pathogens, enabling point-of-care diagnostics. The identified proteins confidently detected within the three biological replicates for each strain included numerous virulence factors and carbohydrate-metabolizing enzymes, which have been previously identified as discriminative markers for the identification of *S*. *pneumoniae*.^24,62^ Using subcellular localization tools (PsortB and DeepLocPro),^60,61^ it was possible to corroborate *in silico* the surface localization of the proteins. The *S*. *pneumoniae* strain TIGR4 is well characterized^53^ and thus its proteome was used to compare the identified proteins among the 11 different analyzed strains.

Surface proteins are important targets in bacteria,^72^ because they interact with the surrounding environment during infections,^17,73^ and thus are investigated as targets for improving diagnostics,^62,74^ generating protein-based vaccines,^38,43,75–77^ therapeutic antibodies and/or pathoblockers.^18,78,79^ Different protein-based vaccines have been developed against *S*. *pneumoniae*,^3,18,80–83^ to prevent the serotype replacement observed with the use of pneumococcal conjugate vaccines (PCVs),^3,76,84,85^ highlighting the need for constant identification of surface protein targets. To find the mentioned targets, different approaches of surface-shaving of intact bacteria have been employed.^35,39–45,86^ More specifically, surface-shaving of *S*. *pneumoniae* has revealed promising surface proteins that were used to build an array for diagnosing pneumococcal infections.^62^ Through LPI surface-shaving, it was possible to validate and identify interesting protein targets consistently present among the studied strains (Figure 3; Supporting Information 10). As previously shown, the LPI approach, for performing surface-shaving of intact bacteria (Nanoxis Consulting AB, Gothenburg, Sweden), has demonstrated the capability of short and controlled digestion times and high reproducibility between replicates.^42,44^ Given the nature of the samples low intensities in mass spectra were expected, therefore, the achieved coverage of the theoretical proteomes was low in comparison to other surface-shaving experiments (20%–30%).^69^ However, relevant surface proteins which have been described as antigens^62^ and epitopes^64^ for *S*. *pneumoniae* were found in most of the analyzed replicates.

### Potential Novel Biomarkers for *Streptococcus pneumoniae*

From our surface-shaving experiments, 34 proteins were identified as potential novel *S*. *pneumoniae* biomarkers (Supporting Information 9), many of which exhibited interspecies similarity, with closely related species likely due to shared ancestry between *S*. *pneumoniae* and *S*. *mitis*, which share between 60% to 80% of their orthologous genes.^24,87,88^ Proteins like peptidoglycan lytic transglycosylase MltG, UTP-glucose-1-phosphate uridylyltransferase Cap4C and 1,4-beta-N-acetylmuramidase, lysozyme LytC, present in other Gram-positives, were very prevalent in our proteomics experiments and in our *in silico* analysis; therefore, even when out of our scope, we consider these targets as an interesting “broad-spectrum” countermeasure for treatments against Gram-positive bacteria. Abundance levels were also estimated in this work, enabling phenotypic comparison between the selected strains and, thus, highlighting potential biomarkers that were conserved for *S*. *pneumoniae* and were also present in a majority of strains.

### Biological Relevance of the *Streptococcus pneumoniae* Species-Specific Proteins

Proteins from the protein groups described by Aceil and Avci, 2022,^18^ cholesterol-dependent cytolysins, choline-binding proteins (CBPs) and pneumococcal histidine triad proteins (PHTs) together with “moonlight” and ribosomal proteins^71^ were classified as surface biomarker candidates. CBPs are surface-associated virulence factors anchored via phosphorylcholine in teichoic acids^72,89^ and multiple prominent CBPs were identified across our analyzed strains. They mediate adhesion, immune evasion, biofilm formation, and autolysis.^90–92^ PspA inhibits complement and binds host molecules like lactoferrin^29^ and LDH,^75^ while CbpA/PspC promotes epithelial translocation and immune evasion via interactions with IgA, pIgR, and factor H.^92–94^ Autolysins LytA–C support peptidoglycan remodeling and biofilm growth; LytA also releases pneumolysin and degrades C3b/iC3b,^95^ while LytB enhances complement deposition.^96^ Several CBPs are being evaluated in combination vaccines to broaden serotype coverage^75^ or as enzybiotics.^89^

Pneumococcal histidine triad proteins (PHTs), including PhtA–E, are conserved, surface-exposed and metal-binding via HxxHxH motifs, contributing to colonization and immune evasion.^38,97–100^ PhtA protects across serotypes and in murine models, while PhtE is the most divergent and immunogenic, reducing adherence and colonization *in vivo*.^101–103^ PhtD has been used in phase II trials both as a monovalent and multivalent vaccine antigen.^76,104^

Extracellular glycosidases such as neuraminidases (e.g., NanA), β-galactosidases (e.g., BgaA), and N-acetylglucosaminidases (e.g., StrH) cleave host glycans to support colonization, nutrient access, and immune evasion.^36,70^ These enzymes reduce complement activity and enhance persistence. In our data set, these glycosidases were highly abundant. NanA promotes adhesion, biofilm formation, complement resistance, and blood–brain barrier penetration,^105–109^ although biomarker alignment required substitution of the TIGR4 sequence due to low similarity. BgaA supports colonization and inhibits complement opsonophagocytosis,^36,105,110–113^ while StrH removes terminal GlcNAc, further aiding immune evasion.^105,114^ Zinc metalloproteases (ZmpA–D) mediate colonization, immune modulation, and inflammation.^30,115,116^ ZmpA cleaves IgA1 to inhibit Fc-mediated immunity while preserving Fab adherence,^116,117^ and ZmpB, the most widespread Zmp, induces inflammation and reflects ancient mucosal adaptation.^116^

Proteins such as PavA enolase, GAPDH, 6-phosphogluconate dehydrogenase, serine protease, and phosphoglycerate kinase, that were found across all our different comparisons, highlight the importance of so-called nonclassical surface proteins^72^ as surface biomarker candidates; that, due to a lack of a characteristic signal peptide or membrane anchor motif, are poorly classified by subcellular localization tools. Different ribosomal proteins have been described to be involved in processes like antimicrobial and chaperone activities, additionally to their functions transcription, translation and regulation of gene expression.^71,118^ Furthermore, its presence in cytotoxic *S*. *pneumoniae* extracellular vesicles,^119^ suggests that their localization is not limited to the cytoplasm.

### Species-Specific Proteins to Identify *Streptococcus pneumoniae*

In 2015, Olaya-Abril et al. described a list of antigens that were used for the identification of *S*. *pneumoniae*. In our data set, 14 antigens of those described by Olaya-Abril et al., 2015 ^62^ were identified, glycosidases such as beta-galactosidase; immunoglobulin A; and glucosaminidase, were the most prevalent proteins found in most of our replicates (Table 2). Glycosidases are, interestingly, surface proteins in charge of carbohydrate digestion when *S*. *pneumoniae* colonizes host cells.^17,36,105^ On the other hand, the species-specific *S*. *pneumoniae* biomarkers were confirmed based on the genomic work performed by Kilian and Tettelin, 2019,^24^ wherein 224 unique genes predicted, we identified 25 proteins, some of which were detected on the bacterial surface and had virulence factor properties (Table 3).

### Species-Specific Peptides to Identify *Streptococcus pneumoniae*

Proteotyping, developed over the past 15 years, uses mass spectrometry to identify unique peptides for microbial species, strains, or traits like antibiotic resistance and virulence.^42,44,45^ It distinguishes closely related species (e.g., Mitis group *Streptococcus*) and strain-level variation (e.g., *Helicobacter pylori*).^42,45^ In combination with Microorganism Classification and Identification (MiCId), and a taxonomically curated database;^66^ we identified several potential peptide biomarkers, unique for *S*. *pneumoniae*. Interestingly, many of the unique peptide biomarkers are from the cell surface or cell wall proteins with a virulence factor role, such as BgaA, NanA, StrH, and Iga.^108,109,111–115^ Of high interest, general stress protein 24 was present in all strains and all replicates and from which two peptides were top ranked. Moreover, in a previous study,^120^ where patient samples were analyzed directly by tandem mass spectrometry, the peptide “VSD­VAE­STG­EFT­SEQ­FEK” was described as a relevant biomarker for *S*. *pneumoniae*, thus confirming our findings. Biomarker peptides, generated from the LPI-based surface-shaving approach, could be suitable for detection of the bacterial surface, by means of antibodies or other molecular receptors. These peptides can be viewed as exposed linear epitopes which have previously been successfully used for generating antibodies.^42,121^

### Limitations of the Study

Among the limitations of our study, we acknowledge that the analysis of *in vitro*-cultured strains may not reflect the actual presence *in vivo*, that is, during an infection situation. Furthermore, differences in growth conditions such as media, and/or growth phase will impact the expression and therefore the abundance of proteins. As shown before, the protein relative abundance and thus the abundance of biomarkers, varies when analyzing patient samples as compared to cultures,^120^ which should be considered upon further characterization of the proposed biomarker candidates. We acknowledge that although some proteins demonstrate high sequence similarity (>90%), others are very variable between the strains, such as the LysM, PhtE, and MurM/FibA proteins. Some virulence factors, such as the PspA, may even belong to different families. These considerations are relevant when designing diagnostic tools or protein-based vaccine or therapeutics.^32,81^

## Conclusions

This study presents a comprehensive proteomic analysis of *Streptococcus pneumoniae*, integrating phylogenomic selection of 11 genetically diverse strains with optimized surface-shaving and LC-MS/MS proteomics. Surface accessible proteins were identified by combining this approach with comparative genome analyses and taxonomic confirmation, revealing a conserved core proteome as well as strain-specific features. The bioinformatic analyses identified 34 proteins as potential novel *S*. *pneumoniae* biomarkers, many of which were present across the 11 strains. These species-specific proteins could be relevant for diagnostics and theraputic measures.

Surface-shaving identified numerous noncytoplasmic proteins, including well-known virulence factors, such as NanA, BgaA, and ZmpA, as well as novel candidates such as SP_0097 and SP_1804, from which limited information can be retrieved. However, similar proteins are part of the pneumococcal membrane and are consistently found in *S*. *pneumoniae*. Moreover, cross-reference with published biomarker data sets confirmed the presence of 81 previously presented surface proteins and 25 species-specific proteins.^24^

Proteotyping, using MiCId, identified multiple *S*. *pneumoniae*-unique peptides, further supporting their diagnostic potential. Many of these species-specific proteins and peptides are surface-localized and conserved, highlighting their suitability for serotype-independent vaccine and therapeutic development. Despite the limitations inherent to *in vitro* studies, the consistent abundance of the identified proteins and peptides across strains and replicates provides strong evidence for their biological relevance. Future studies should explore *in vivo* abundances and functionality of these targets, contributing knowledge in the urgent search for novel therapeutic targets due to the increase in the global spread of antibiotic resistance.

## Supplementary Material





## Data Availability

The mass spectrometry proteomics data have been deposited to the ProteomeXchange Consortium via the PRIDE^122^ partner repository with the data set identifier PXD063486 and 10.6019/PXD063486.

## Notes

Descriptions of the content on the spreadsheets (PDF)
*Streptococcus pneumoniae* strain characteristics, *Streptococcus pneumoniae* strain selection, unique protein biomarkers (224) in NCBI genomes, unique protein biomarkers (224) in 11 strains, proteomic information and functional annotation of the identified proteins by surface-shaving proteomics, label-free quantification of the proteins detected by surface-shaving and tandem mass spectrometry, surface-shaving optimization conditions for LPI, common proteins detected in all the replicates of the 11 strains, *Streptococcus pneumoniae* potential biomarkers confirmed in silico, relative protein abundance of all proteins found in 11 *Streptococcus pneumoniae* strains, and *Streptococcus pneumoniae* potential protein biomarkers based on the relative abundances (XLSX)

## Abbreviations

LPI: Lipid–protein immobilization
LC-MS/MS: Liquid chromatography tandem mass spectrometry
CCUG: Culture collection university of Gothenburg
PBS: Phosphate buffered saline
BLAST: Basic local alignment search tool
PSM: Peptide spectrum match
HCD: Higher-energy collisional dissociation
MiCId: Microorganism classification and identification
LFQ: Label free quantification
ANIb: Average nucleotide identity based on BLAST
GGDC: Genome to genome distance calculator
NCBI: National Center for Biotechnology Information
PCV: Pneumococcal conjugate vaccine
CBPs: Choline binding proteins
PHTs: Pneumococcal histidine triad proteins
OD: Optical density
FRD: False rate discovery
GO: Gene ontology
ppm: Parts per million
