# Supplementary material for: Identification of Streptococcus pneumoniae -Specific Proteins by Surface-Shaving Proteomics
Source: J Proteome Res. 2025 Nov 10;24(12):6154–73. doi: 10.1021/acs.jproteome.5c00716 (PMC12687362; doi:10.1021/acs.jproteome.5c00716)
Supplement: Supplementary file 1 [file pr5c00716_si_001.pdf]

Supporting Information

Identification of *Streptococcus pneumoniae* Specific Proteins by Surface-Shaving Proteomics

Leonarda Acha Alarcon ,[†,‡] *Guillem Seguí* ,[‡,§,ǁ] *Beatriz Piñeiro-Iglesias* ,[‡,§,ǁ]

Ema Svetlicic ,[⊥] *Nahid Kondori* ,[§] *Margarita Gomila* ,[#] *Edward R. B. Moore* ,[†,‡,§,ǁ]

and Roger Karlsson [*,†,‡,§,∇]

[†] Department of Infectious Diseases, Institute of Biomedicine, Sahlgrenska Academy, University of Gothenburg ,40530 Gothenburg, Sweden.

[‡] Centre for Antibiotic Resistance Research (CARe), University of Gothenburg , 40530 Gothenburg, Sweden.

[§] Department of Clinical Microbiology, Sahlgrenska University Hospital , Region Västra Götaland, 41345 Gothenburg, Sweden.

[ǁ] Culture Collection University of Gothenburg (CCUG), Sahlgrenska University Hospital and Sahlgrenska Academy , 41390 Gothenburg, Sweden.

[⊥] Novo Nordisk Foundation Center for Biosustainability, Technical University of Denmark , 2800 Kongens Lyngby, Denmark.

[#] Microbiology (Biology department), University of the Balearic Islands , Ctra. de Valldemossa km 7,5, 07122 Palma de Mallorca, Spain.

[∇] Nanoxis Consulting AB , 40016 Gothenburg, Sweden.

[*] email: roger.karlsson@gu.se .

Table of contents

Supporting Information 1. *Streptococcus pneumoniae* strain characteristics.

Supporting Information 2. *Streptococcus pneumoniae* strain selection.

Supporting Information 3. Unique protein biomarkers (224) in NCBI genomes.

Supporting Information 4. Unique protein biomarkers (224) in 11 strains.

Supporting Information 5. Proteomic information and functional annotation of the identified proteins by surface-shaving proteomics.

Supporting Information 6. Label free quantification of the proteins detected by surface-shaving and tandem mass spectrometry.

Supporting Information 7. Surface-shaving optimization conditions for LPI.

Supporting Information 8. Common proteins detected in all the replicates of the 11 strains.

Supporting Information 9. *Streptococcus pneumoniae* potential biomarkers confirmed *in silico.*

Supporting Information 10. Relative protein abundance of all proteins found in 11 *Streptococcus pneumoniae* strains.

Supporting Information 11. *Streptococcus pneumoniae* potential protein biomarkers based on the relative abundances.
